# Alcohol Policies Affect Drinking Patterns—A Potentially New and Harmful Drinking Pattern in Consumers of Small Vodka Bottles (SVB) in Poland

**DOI:** 10.3390/ijerph192417047

**Published:** 2022-12-19

**Authors:** Andrzej Silczuk, Małgorzata Mańczak, Jakub Owoc

**Affiliations:** 1 Department of Public Health, Medical University of Warsaw, 02-091 Warsaw, Poland; 2 Department of Prevention and Treatment of Addictions, Institute of Psychiatry and Neurology, 02-957 Warsaw, Poland; 3 National Institute of Geriatrics, Rheumatology, and Rehabilitation in Warsaw, 02-637 Warsaw, Poland

**Keywords:** alcohol use, drinking patterns, vodka mini-bottles

## Abstract

Drinking alcohol has a vast and diverse impact on many aspects of people’s lives around the world. It is a major public health concern and is subject to numerous legal regulations and limitations. So far, little attention has been paid to if and how the volume of alcohol containers may affect drinking patterns. The widespread availability in recent years in Poland of small vodka bottles in various flavors and sizes was the rationale behind investigating whether the phenomenon may affect drinking patterns in any way. This was a 12-month cross-sectional survey study that started in January 2020. It included a total of 217 inpatients and outpatients that met the ICD-10 alcohol dependence criteria. The respondents were asked about their drinking habits and the use of small vodka bottles. It was found that respondents who regularly use small vodka bottles were much more likely to start their drinking early in the morning. The widespread availability and selection of small vodka bottles may encourage and facilitate drinking that starts early in the morning. It also makes it easier to maintain and control intoxication throughout the whole day, which could be considered another drinking pattern different from the other well-established ones, such as binge or continuous drinking. However, the design of this study makes it impossible to draw firm conclusions and further research is necessary.

## 1. Introduction

Alcohol has played an important social role in almost all cultures for millennia. This role has always come at a price. Excessive drinking and its consequences are a thoroughly studied and recognized problem in socioeconomic and medical terms. According to the WHO, alcohol is a causal factor in over 200 diseases and is responsible for 5.3% of all deaths worldwide, or even 13.5% in younger age groups [1]. Meanwhile, the costs of excessive drinking, such as lost productivity, medical treatments, law enforcement, or motor vehicle incidents, may amount to as much as USD 249 billion a year in the United States alone [2]. Unsurprisingly, this vast impact has long been of concern to public authorities and is subject to numerous and diverse legal regulations to minimize its negative effects. The most common policy options usually focus on restrictions on the availability of alcoholic beverages, such as minimum age, price, and retail sales regulation. Studies show that these measures tend to be successful, as opposed to education or public information campaigns [3]. However, history shows that total prohibition is not a viable solution, as substantial social demand promotes the informal market and organized crime. Thus, national policies are typically a mix of various measures that significantly depend on local and cultural aspects.

The impact of alcohol consumption on health outcomes is largely determined by two separate but related dimensions, the volume of alcohol consumed and drinking patterns [1], which include all aspects of drinking not covered by the term “volume” [4]. Patterns typically refer to the frequency of drinking; however, as there are too many individual factors involved, it is difficult to define universally accepted and objective types of patterns. Some criteria focus on frequency and volume (standard drinks in a period of time: moderate, binge, extreme, heavy) [5], while others also put some emphasis on less objective criteria such as place (e.g., home or public places) or context (e.g., with meals or special events). 

The three decades of a free market economy in Poland have witnessed a gradual shift toward a western model of alcohol sales with an increasing share of beer and wine along with a shrinking share of spirits. Nevertheless, binge drinking was and still is considered the dominant pattern of drinking in society [6,7]. Recent years have seen some changes in that trend as spirits producers focus more on flavored and colored vodka sold in small bottles (such as 100 mL and 200 mL, or 3.4 oz or 6.8 oz, respectively) and their widespread availability and exposition at points of sale. This strategy resulted in a significant sales increase in small vodka bottles (SVB) [8].

Meanwhile, common observations at shops or media and market reports [15—synergion] suggest that a substantial part of this increase refers to early hours on weekdays rather than evenings and weekends. Consumers apparently appreciate the merits of an affordable (small size, lower price), convenient (easy to hide, a screwcap allowing them to save some for later), and easy-to-drink (sweet flavor) alcoholic drink. Clinical interviews conducted with alcohol-dependent patients as a part of one of the author’s (AS) everyday work and press reports suggest that SVBs may play a growing role in the behaviors of alcohol consumers. 

Sizes of alcohol bottles are usually not subject to official regulations, leaving much room for creativity and marketing techniques among producers. Meanwhile, many countries such as the United States [9] and European Union [10] have established a minimum number of cigarettes in a single pack at 20 to make them less affordable for young people. Thus, it is likely that small and affordable spirits with sweet flavors attract young consumers; however, we lack data to support that. Nevertheless, the size of alcohol bottles may be an important public health and policy issue in many aspects and should be investigated more thoroughly. Similar observations have been made on the subject of the glass shape and its possible influence on drinking behaviors [11], and the glass shape appeared to influence the rate of drinking alcoholic beverages and became a modifiable target for public health interventions [12]. That is a reasonable rationale to focus on the size of the high percentage of alcoholic beverages sold—SVBs—as a possible and unstudied threat to public health in the domain of dependencies, as well as the possible impact on drinking patterns. Both of the above became hypotheses that the authors have made by designing this simple and feasible study in clinical settings. 

Meanwhile, in Poland in the recent two decades, some phenomena have appeared. As 25% of premature deaths in Poland are alcohol-related, alcohol consumption almost doubles in 10-year periods [13], and some hypotheses have been raised that this may have a direct impact on life expectancy [14].

We hypothesize that a new pattern of drinking may be emerging, one in which consumers drink smaller portions but more often and throughout the day. The main objective of this study was to investigate the drinking patterns of alcohol-dependent patients with a special focus on their use of SVBs and time of consumption. So far, there has been very little evidence on how the size of bottles may affect drinking. This study may be the first scientifically alarming “red flag” to encourage research and voices to provide interventions in public health and changes in alcohol policies. 

## 2. Materials and Methods

This was a cross-sectional study carried out for 12 consecutive months starting in January 2020 on a sample of alcohol-dependent patients at the Institute of Psychiatry and Neurology in Warsaw in two wards: the Detoxification Ward for patients hospitalized for alcohol withdrawal syndrome (AWS) (drinking cessation happened between 12–72 h) and the Rehabilitation Therapy Ward (drinking cessation happened at least 73 h prior to the hospitalization) for patients with alcohol dependence (voluntary treatment for less-damaged-by-alcohol inpatients in an open setting—the patients participated in whole-day group sessions but slept at home). The following inclusion criteria were used: age 18–65 years, informed consent to participate in the study, and meeting criteria for alcohol dependence according to the ICD-10 research criteria for F10.2 [15]. Exclusionary criteria were: patients diagnosed with the use of or dependence on drugs (for nonmedical reasons) such as benzodiazepines, nonbenzodiazepine hypnotics, opioids, and medications registered as drugs in the treatment of alcohol dependence; unable to participate in the study due to their mental states (consciousness disorders, significant cognitive impairment, inability to collect reliable data). 

The respondents were interviewed by psychiatrist using a structured questionnaire that also included the Alcohol Use Disorders Identification Test (AUDIT) [16,17]; criteria for alcohol dependence according to ICD-10; and questions referring to the characteristics of the patients, their drinking habits (type, time, frequency, volume, active recovery, abstinence), course of the disease and treatment, or the use of other substances. All respondents had laboratory tests performed as a part of a routine practice upon admission.

This research project obtained the consent of the local Bioethics Commission. 

## 3. Statistics

Statistical analysis was performed using Statistica, version 13 (StatSoft Inc., Tulsa, OK, USA). Decisions on the selection of appropriate statistical tests in subsequent analysis steps were based on the results of tests for compliance with the normal distribution: Kolmogorov–Smirnov (for *N* > 100) and/or Shapiro–Wilk (for *N* ≤ 100). To check the intergroup differences, we used a parametric Student’s *t*-test for independent samples, as well as a nonparametric U Mann–Whitney test. The χ2 test or the χ2 test with Yates correction was used for comparisons of nominal variables. Univariate and multivariate logistic regression models were used to evaluate predictors of SVB use. A *p*-value of less than 0.05 was considered statistically significant.

## 4. Results

The study involved 217 alcohol-dependent patients: 170 (78%) men and 47 (22%) women. Two-thirds of the respondents were patients of the Detoxification Ward and one-third of the respondents were patients of the Rehabilitation Ward. For the majority of respondents, the main alcohol they usually drink is vodka (75%). Only 22% of them drink mainly beer, and 3% mainly wine. Most alcohol-dependent patients (73%) use small vodka bottles. Table 1 shows a comparison between patients who use SVB and those that do not. The statistically significant differences between them refer to only two dimensions: SVB users smoke cigarettes more often (*p* = 0.018) and start drinking alcohol earlier in the morning (*p* < 0.001). 

A univariate and multivariate logistic regression analysis was performed. In the univariate analysis, the odds of using SVB were found to be twice as high in a smoking patient as in a nonsmoking patient (OR = 2.09; 95%CI: 1.13–3.38). The earlier patients start drinking, the higher the chances of using SVB: if drinking starts before 7 a.m., the chance of using SVB is over five times higher than among patients who start drinking after 5 p.m. (OR = 5.71; 95%CI: 2.19–14.89). In the multivariate analysis, smoking is no longer statistically significant, and early drinking is still significant. If someone starts drinking before 7:00 a.m., the chance of using SVB is 5.5 times higher than for a respondent who starts drinking after 5:00 p.m. (Table 2).

## 5. Discussion

The evidence on how the size of alcohol containers affect drinking is very scarce. The only identified study concluded that providing households with smaller wine bottles (50cl instead of the usual 75cl) did decrease consumption [18]; however, wine is subject to different drinking patterns than spirits. This finding is in line with numerous other studies related to food and nonalcoholic drinks. The 2015 comprehensive Cochrane systematic review found that people tend to consume more food and drink when offered larger-sized portions and suggested that policy options and practices aimed at reducing the size of packages may contribute to the reduction in consumption. The review was not able to identify any alcohol-related studies. Interestingly, there has been more research into how glass shape affects the consumption of alcoholic beverages [12] or the amount of alcohol poured [19], providing evidence that even small details may affect drinking habits. 

Our results and sample suggest a different and potentially damaging pattern when it comes to smaller containers and spirits. 

The major finding in this study is that 73% of all participants use SVBs and the vast majority of that group (83%) starts drinking before 12 a.m., with most of them even as early as 7 a.m. Although these are the most statistically significant findings and associations in the entire study, they raise more questions than they provide answers due to the cross-sectional design and unique sample. The questions that naturally arise are whether we are dealing with a pattern of drinking in which drinkers sustain a low-to-moderate level of alcohol in their blood from early morning and attempt to function normally. As the sample included diagnosed, alcohol-dependent individuals seeking professional help, another consideration is what was the role of SVBs in the damaging process that led to it? Did SVBs, which provide easy, quick, affordable, and controllable drinks, actually lead to it or just facilitate the inevitable? Assuming there are at least indications that it may be, to some extent, the case, the public health policy concern is whether the widespread availability and exposition of SVBs may be hazardous for the public. 

The lack of scientific evidence and research makes a comprehensive discussion challenging and requires hypotheses, gray literature, market data, and legal regulations to rely on. During our daily clinical work in recent years, we have learned that more and more patients started drinking early in the morning and frequently used SVBs for that purpose. The most popular sizes of SVB offer 100 mL or 200 mL of spirits containing 30–40% alcohol, the equivalent of 2.3 or 4.6, respectively, standard units of alcohol according to the U.S. norm [20]. This is far more alcohol than a beer (one standard unit), and an SVB can be drunk quickly due to its volume and sweet flavor or saved for later as it is easy to conceal bottles with a screwcap. The available sales data from Poland suggest that the share of SVBs has been on the rise and that SVBs have been a dominant size in the flavored vodka market [8]. Meanwhile, other market segments of spirits, such as pure vodka or whiskey, are dominated by larger-sized containers (0.5 L or more). The results from our study that suggest SVBs are typically drunk early in the day are confirmed by non-peer-reviewed research results published in 2019 in Poland [21]. The estimations based on two separate surveys of 150 stores and 1000 respondents (drinkers) suggested that app. 30% of all SVB sales take place by 12.00 a.m. The report gained widespread media attention, and soon after it was published, authorities decided to increase the taxation of vodka bottles smaller than 300 mL in an attempt to counteract the ubiquity of SVBs [22]. Another instance of legal regulations on SVBs comes from the state of Utah which banned, in 1990, sales of 1.75 oz bottles of alcohol to protect underage drinkers, among other reasons [23]. Nevertheless, examples of SVBs as public health concerns remain limited. 

## 6. Conclusions and Limitations

The results and other available data may indicate that SVBs may lead to a previously unrecognized drinking pattern of consuming small quantities of alcohol throughout the day to control and maintain a state of intoxication. The unrepresentative and specific sample of diagnosed alcoholics should be considered a limitation, yet its specificity suggests that this hypothetical pattern may have very serious social and health consequences. Other limitations include the cross-sectional design of the study, a self-reporting bias among respondents, and the local character of the study, as drinking patterns may vary significantly between countries. The main strength is that the perspective of SVB consumption has never been subject to any research. This study was conducted based on available clinical resources in order to identify a certain phenomenon in people experiencing serious alcohol-related harm. We would definitely recommend and think about designing a more in-depth investigative study to compare groups that drink moderately or periodically. There is no question that the surprising rise in the sales of small vodka bottles does not only come from alcohol-dependent individuals (estimated to account for about 4.4% of the adult Polish population [24]). Future studies should investigate in more depth drinking patterns for SVBs, motivations for using them, quantities, and whether and how they may contribute to the ultimate loss of control over one’s drinking. One may also hypothesize that lockdowns and remote work, in particular, caused by COVID-19 waves can contribute to increased SVB consumption because they facilitate controlled drinking during working hours. Some studies already suggest that the pandemic caused an upsurge in drinking in the U.S. [25].

We would like to consider this study a preliminary one in order to initiate discussion on the topic of how the widespread availability of SVBs may inflict harm. Perhaps increasing prices through taxation—effective in the case of cigarettes [25,26,27]—or even prohibiting its sales could benefit public health. Further research is recommended because alcohol container sizes are an easily modifiable target for public health interventions, and the study results are the first “red flag” for public considerations of limiting the sale of SVBs.

## Figures and Tables

**Table 1:** Characteristics of respondents and their drinking habits.

	SVB Users *n* = 159	Not SVB Users *n* = 58	*p*
Age, Me (IQR)	45 (37–54)	45 (39–57)	0.311
sex			
men	129 (81%)	41 (71%)	0.098
women	30 (19%)	17 (29%)	
education			
elementary	16 (10%)	4 (7%)	0.447
secondary	106 (67%)	36 (62%)	
tertiary	37 (23%)	18 (31%)	
self-assessment of drinking			
moderate	21 (13%)	12 (21%)	0.174
heavy	138 (87%)	46 (79%)	
time of first alcohol intake			
before 7:00 a.m.	79 (50%)	15 (26%)	<0.001
7:00 a.m.–12:00 p.m.	55 (35%)	20 (34%)	
12:00– 5:00 p.m.	13 (8%)	10 (17%)	
after 5:00 p.m.	12 (8%)	13 (22%)	
cigarettes			
yes	92 (58%)	23 (40%)	0.018
no	67 (42%)	35 (60%)	
Did you drink more in the last year?			
less	21 (13%)	9 (16%)	0.840
more	106 (67%)	39 (67%)	
no changes	32 (20%)	10 (17%)	

**Table 2:** Univariate and multivariate analyses.

	Univariate Analysis		Multivariate Analysis	
	OR (95% CI)	*p*	OR (95% CI)	*p*
time of the first alcohol intake				
before 7:00 a.m.	5.71 (2.19–14.89)	<0.001	5.56 (2.11–14.67)	0.001
7:00 a.m.–12:00 p.m.	2.98 (1.17–7.60)	0.022	3.11 (1.20–8.04)	0.019
12:00–5:00 p.m.	1.41 (0.45–4.40)	0.555	1.63 (0.51–5.23)	0.410
after 5:00 p.m.	ref			
cigarettes				
yes	2.09 (1.13–3.86)	0.019	1.88 (0.99–3.58)	0.055
no	ref			

## Data Availability

Not applicable.
